# Mitochondria: Central Organelles for Melatonin′s Antioxidant and Anti-Aging Actions

**DOI:** 10.3390/molecules23020509

**Published:** 2018-02-24

**Authors:** Russel J. Reiter, Dun Xian Tan, Sergio Rosales-Corral, Annia Galano, Xin Jia Zhou, Bing Xu

**Affiliations:** 1 Department of Cellular and Structural Biology UT Health San Antonio, San Antonio, SD 78229, USA; tan@uthscsa.edu (D.X.T.); ZhouX4@uthscsa.edu (X.J.Z.); doctxu@126.com (B.X.); 2 Centro de Investigacion Biomedica de Occidente, Instituo Mexicana del Seguro Social, Guadalajara 44346, Mexico; espiral17@gmail.com; 3 Departamento de Quimica, Universidad Autonoma Metropolitana-Iztapatapa, Mexico D.F. 09340, Mexico; agalano@prodigy.net.mx

**Keywords:** oxidative stress, free radicals, electron transport chain, oxidative phosphorylation, free radical theory of aging, melatonin uptake, melatonin synthesis

## Abstract

Melatonin, along with its metabolites, have long been known to significantly reduce the oxidative stress burden of aging cells or cells exposed to toxins. Oxidative damage is a result of free radicals produced in cells, especially in mitochondria. When measured, melatonin, a potent antioxidant, was found to be in higher concentrations in mitochondria than in other organelles or subcellular locations. Recent evidence indicates that mitochondrial membranes possess transporters that aid in the rapid uptake of melatonin by these organelles against a gradient. Moreover, we predicted several years ago that, because of their origin from melatonin-producing bacteria, mitochondria likely also synthesize melatonin. Data accumulated within the last year supports this prediction. A high content of melatonin in mitochondria would be fortuitous, since these organelles produce an abundance of free radicals. Thus, melatonin is optimally positioned to scavenge the radicals and reduce the degree of oxidative damage. In light of the “free radical theory of aging”, including all of its iterations, high melatonin levels in mitochondria would be expected to protect against age-related organismal decline. Also, there are many age-associated diseases that have, as a contributing factor, free radical damage. These multiple diseases may likely be deferred in their onset or progression if mitochondrial levels of melatonin can be maintained into advanced age.

## 1. Introduction

A surplus of chemically-reduced oxygen derivatives, often referred to as reactive oxygen species (ROS), some of which are free radicals (with an unpaired valence electron), commonly leads to an augmented level of molecular damage identified as oxidative stress [1]. The excess of highly reactive oxygen metabolites overwhelms a complex antioxidant defense network such that it does not adequately defend against the consequent deleterious effects. All major molecular groups typically sustain damage when attacked by free radicals, but the level of oxidative stress is most frequently based on the quantities of damaged lipid products, protein carbonyls, and mutilated nucleic acids [2]. While many of the toxic derivatives of ground state oxygen are oxygen-based and therefore are referred to as reactive oxygen species (ROS), others are nitrogen (RNS) or chlorine (RCS)-based. For the purposes of the current report, these are all considered under the collective term of ROS. Likewise, the damage inflicted by ROS, depending on the species involved, is referred to as either oxidative stress or nitrosative stress. Herein, both are categorized as oxidative stress.

The generation of ROS, including free radicals, is inevitable and continuous in aerobic organisms [3]. Since their creation cannot be totally smothered, the alternative is to neutralize them before they vandalize a neighboring critical molecule. This is obviously not a foolproof task for cells, however, since invariably some radicals escape incapacitation and destroy bystander molecules, thereby compromising organellar physiology. Less than optimal functioning of subcellular systems can undermine cellular metabolism leading to physiological inefficiency and, in the worst case scenario, cell death [4].

To make matters worse, once formed, free radicals can cause a chain reaction of events that leads to massive molecular annihilation of healthy structures. This occurs when the first free radical formed extracts an electron from an otherwise normal molecule in its vicinity, causing it to become a destabilized free radical; in turn, it captures an electron from another molecule to sabotage it. This domino process is especially well defined in reference to lipid peroxidation and is only interrupted when an antioxidant intervenes and scavenges the perpetuating free radical or an intermediate toxic derivative [5]. 

Not all free radicals are pariahs. While under conditions of oxidative stress in non-pathological cells, they produce an imbalance between the reductive power of the cell and the oxidation state in favor of the latter, under some conditions, they serve as beneficial signaling molecules [6,7]. Undoubtedly, ROS have several characteristics typical of second messengers, including their short half-life, as well as the ability to amplify a series of reactions that are initiated by a primary ligand. In particular, O_2_•^−^ and H_2_O_2_ can function as intracellular signaling molecules; this is assisted, especially in the case of H_2_O_2_, by its ability to readily pass through cellular membranes. The production of the superoxide anion radical (O_2_•^−^) and hydrogen peroxide (H_2_O_2_) are carefully regulated intracellularly [8,9]. This, however, does not apply to the hydroxyl radical (•OH). Finally, ROS and free radicals are sometimes produced in abundance in cancer tissues that aid in the killing of these disease-causing cells [9]. A notable conundrum that becomes apparent when considering the differential actions of ROS and free radicals is how antioxidants/free radical scavengers discriminate between those that are potentially harmful while mostly sparing those mediating beneficial signal transduction processes [10]. Even when very large amounts of an antioxidant are given, the signaling pathways that utilize ROS seem to be left functionally intact.

## 2. Melatonin Origin and Distribution

While there are many molecules that potentially function as free radical scavengers, in the current survey, we only summarize the actions and mechanisms of melatonin and its metabolites in terms of their ability to forestall the cellular damage associated with an excess of ROS. Because of its presence in bacteria [11,12], which evolved several billion years ago, we have speculated that melatonin is phylogenetically the oldest antioxidant in existence [13]. Considering its longevity, throughout evolution melatonin has had ample opportunity to hone and diversify its functions in its quest to vanquish toxic oxygen derivatives [14,15], as well as to inherit other functions [16,17,18]. In addition to its presence in all taxa of the animal kingdom (where attempts to measure it have been made), its discovery in land plants [19,20], along with its verification and functional definition in many plant species [19,20,21,22], suggests there is no organism on Earth that lacks this important molecule. Melatonin (and its metabolites) in plants have many of the same functions as this indole has in animals [23,24,25,26].

It has been argued that because of its seemingly lower concentration intracellularly relative to other well-documented antioxidants, e.g., glutathione in neurons and hepatocytes, melatonin would not successfully compete as a free radical scavenger, e.g., of the •OH, which has an extremely short half-life and creates damage only in the immediate vicinity of where it is generated [27]. For any radical scavenger to neutralize the •OH, it is essential that it be at the immediate vicinity of the toxic species. Thus, the total concentration of an antioxidant within a cell may be less important than its concentration at the site of free radical generation. Recently, it was demonstrated that melatonin is in especially high concentrations in mitochondria, an organelle in which free radicals are produced in abundance [28,29]. At least relative to this organelle, melatonin may be “in the right place all of the time” to resist oxidative stress. Thus, melatonin may have a positional advantage that other antioxidants do not share that improves its ability to scavenge toxic radicals and reduce the associated oxidative stress [30]. Judging from its very different concentrations in the bodily fluids of organisms, e.g., blood versus cerebrospinal fluid (CSF) [31], versus ovarian follicular fluid [32], versus bile [33], etc., this may speak to a positional advantage melatonin may have in terms of protecting against oxidative stress. Undoubtedly, any judgement about the levels of melatonin throughout an organism based solely on its concentrations in the blood is erroneous [30].

The specific concentrations of melatonin in a given bodily fluid varies widely depending on a number of factors, e.g., time of day of fluid collection (blood and cerebrospinal fluid (CSF), levels are much higher at night than during the day [31,34]) and the site of fluid collection (melatonin concentrations measured in blood obtained from the vascular sinus surrounding the pineal gland differ from levels in peripherally-collected blood [35,36]); for cerebrospinal fluid (CSF), melatonin levels are higher in this fluid collected from the third ventricle when compared to values in CSF collected via lumbar puncture [31,37]. In reference to ovarian follicles, the fluid of small vesicular follicles has lower melatonin levels than the fluid obtained from large follicles [38]. Moreover, circulating concentrations of melatonin may vary depending on food recently consumed [39], level of stress [40], quality of light exposure [41], age [42], reproductive state [43], use of drugs (medications) [35], presence or absence of disease [44], opacity of the lens in the eye [45], etc.

Melatonin also works via multiple means to limit oxidative stress. While melatonin is capable of directly or indirectly scavenging toxic oxygen species [46,47,48], it has other means at its disposal for combatting free radical damage. When a molecule such as melatonin merely renders one of its delocalized electrons to neutralize a free radical, this action is achieved without receptor intervention [47,49]. It is well documented, however, that melatonin’s ability to limit oxidative stress sometimes also relies on its interaction with melatonin membrane receptors that are present in many, perhaps all, cells [50,51]. These antioxidant actions of melatonin rely on an interaction with membrane receptors located on the cell membrane or on intracellular organelles [52,53,54]; membrane receptors for melatonin also may exist in all organisms [30]. These receptor-mediated actions of melatonin are indirect and likely involve stimulation of antioxidant enzymes, e.g., glutathione peroxidase (GPx), superoxide dismutase (SOD1, 2), SIRT3, etc. [55,56]. When melatonin acts via receptors to carry out its antioxidant actions, it can achieve this effect at much lower concentrations than those required when it functions as a direct free radical scavenger. This relates to the fact that the signal transduction pathways associated with receptors serve to magnify the response. A final feature that characterizes melatonin as an important antioxidant is its availability from multiple sites. Vitamins C and E are only available to humans when they are consumed in the diet. In contrast, given its widespread presence in edible plants [21,26], melatonin is obtained from the food consumed and, furthermore, it is produced in all organisms, perhaps in every cell that has mitochondria or chloroplasts [57,58,59]. As with animals, the concentrations of melatonin in plants vary widely and depend on the plant organ in which melatonin is measured [20,21,60], the physiological state of the plant [26], etc.

Historically, melatonin was thought to be uniquely of pineal gland origin [61,62]. It is now clear that this is not the case, since only vertebrates have a pineal gland, while other animal species and plants lack even a homologous organ. In vertebrates, the circadian synthesis and secretion of melatonin, particularly into the CSF, has a two-fold function; the melatonin rhythm in the CSF is for the purpose of circadian rhythm regulation at the level of the suprachiasmatic nucleus (SCN) and perhaps to protect the brain from oxidative stress [63,64]. However, the pineal gland is not a requirement for the circadian production of melatonin. In the microalga, *Gonyaulax polyhedra* (also known as *Lingulodinium polyedrum*), there is a distinct light:dark-driven melatonin cycle similar to that in vertebrates, but this is a single cell species that obviously has no organs [65].

## 3. Sites of Reactive Oxygen Species Generation

**Figure 1:**
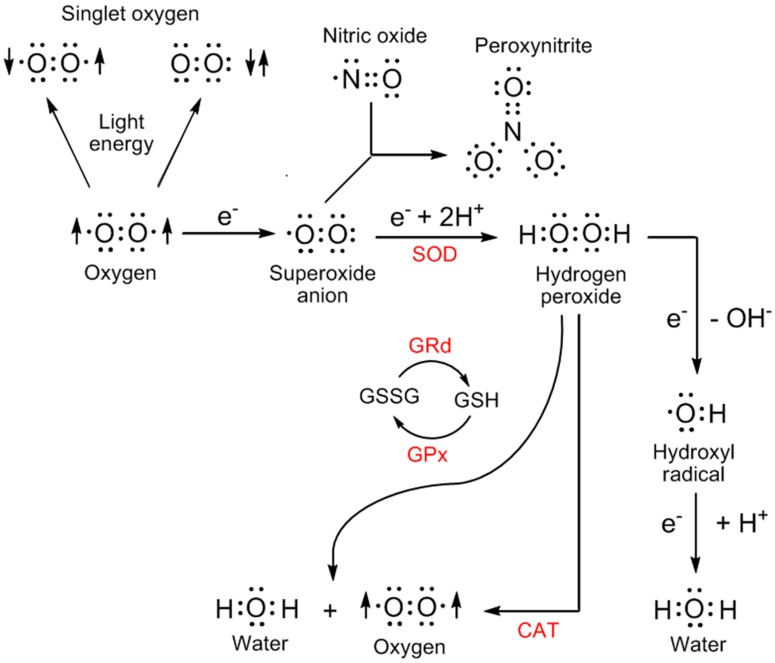
The chemical reduction or the addition of energy to ground state oxygen generates products referred to as reactive oxygen species (ROS). The most reactive of these derivatives are peroxynitrite and the hydroxyl radical. The conversion of hydrogen peroxide to the hydroxyl radical requires a transition metal with the conversion usually being referred to as the Fenton reaction. The red asterisk (*) identifies products that have been reported to be directly scavenged by melatonin and its metabolites. The evidence of these scavenging reactions is much stronger for some ROS than for others. Melatonin also stimulates antioxidant enzymes, e.g., superoxide dismutases (SOD), glutathione peroxidases (GPx), and glutathione reductase (GRd) to indirectly remove toxic ROS. The most toxic species, i.e., peroxynitrite and the hydroxyl radical, are not enzymatically degraded; they can only be removed by a direct scavenger. CAT = catalase.

Mitochondria are a major site for the generation of ROS including free radicals. ROS species, as mentioned in the Introduction, may or may not be damaging to cellular constituents. The oxygen-derived species that have generally low reactivity, e.g., the product of the single reduction of ground state oxygen, the superoxide anion radical (O_2_•^−^), and its enzymatic product hydrogen peroxide (H_2_O_2_) can sometimes serve as second messengers within cells [7,66] while also being precursors of more reactive and destructive species, e.g., the hydroxyl radical (•OH) and peroxynitrite (ONOO^−^), a non-radical but highly oxidizing species (Figure 1). Furthermore, ONOO^−^ may degrade into the •OH [67].

The molecular damage inflicted by especially highly-reactive ROS can be controlled by either preventing the formation of their precursors, i.e., the weakly reactive ROS, or by scavenging them as soon as they are produced. Melatonin has both these capabilities; it reduces O_2_•^−^ formation at the level of the mitochondrial electron transport chain (ETC) by a process referred to as radical avoidance [68], and it is a direct ROS scavenger [15,30,69,70,71] (Figure 1). In addition, melatonin lowers the ROS burden by promoting enzymes that metabolize low reactive ROS to oxygen and water. These enzymes include the superoxide dismutases (SOD1, SOD2), which remove O_2_•^−^, the glutathione peroxidases (GPx), and the peroxiredoxins (PRs), which rids the cellular microenvironment of H_2_O_2_. There are no enzymes that metabolize the most toxic ROS varieties, i.e., •OH and ONOO^−^, to innocuous molecules. While SOD, GPx, and PRs are all known to be regulated by melatonin, the data related to SOD and GPx [72,73,74] is more extensive than that for PRs [75,76]. 

The mechanisms by which melatonin or its metabolites modulate antioxidant enzyme activities have not been unequivocally identified. Some proposed mechanisms include their ability to inhibit the ubiquitination of Nrf2, thereby allowing it to translocate to the nucleus and bind to the antioxidant response element (ARE), which leads to the activation of the associated enzymes [15,77,78]. Melatonin may also modulate SIRT3/SOD2 signaling in the mitochondria to regulate the degree of oxidative stress in this organelle [58].

**Figure 2:**
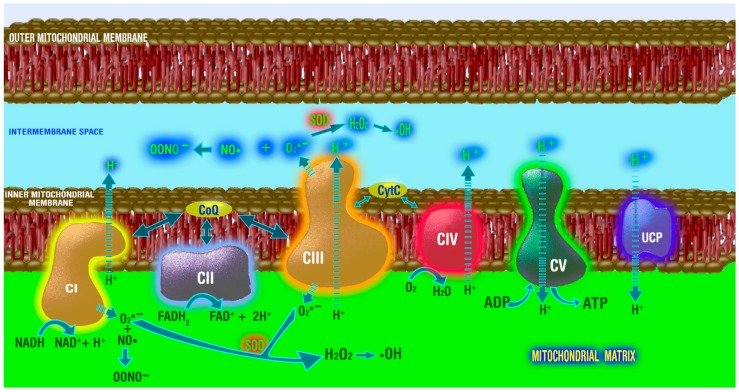
This figure illustrates the structure of a mitochondrion and the location of the complexes (CI-CV) that constitute the electron transport chain that engages in oxidative phosphorylation, which results in the generation of energy in the form of ATP. Free radicals are formed when electrons leak and reduce nearby oxygen (O_2_) molecules to form the superoxide anion radical (O_2_•^−^). CI releases electrons into the mitochondrial matrix, while CIII releases them into both the matrix and the intramembrane space. Once formed, the O_2_•^−^ can be dismutated by superoxide dismutase (SOD) to hydrogen peroxide (H_2_O_2_) with its eventual conversion to the hydroxyl radical (•OH). O_2_•^−^ can also couple with nitric oxide (NO•) to produce the peroxynitrite anion (ONOO^−^). Since melatonin is both taken up and synthesized in mitochondria, it is in an optimal position to scavenge these toxic species.

Within mitochondria, there are numerous sites at which ROS could potentially be generated [79]. The sites that have the greatest potential of doing so under in vivo conditions involve the electron transport chain (ETC). Thus, respiratory Complex I (NADPH: ubiquinone oxidoreductase) and Complex III (ubiquinol: cytochrome c reductase) leak electrons when they are shunted between donor and receptor molecules [80] (Figure 2). The ROS generated by Complex I appear in the matrix, while those from Complex III are divided between the matrix and intermembrane space [81]. Again, while the ROS produced in mitochondria sometimes have physiological relevance [82], their production in excess, as occurs during aging [83,84] and many other pathologies [85,86,87,88,89], has dire consequences due to the damage they inflict. 

While the mitochondrial source of ROS is considered of high significance in terms of disease processes and aging-related cellular and organismal decline, intracellular enzymes outside the mitochondria are also a source of ROS. Some of the major enzymes in this group include monoamine oxidase (MAO), peroxisomal enzymes, NADPH oxidases (NOX), and xanthine oxidase (XO) [76]. NOX, a family of cytosolic enzymes, are of particular importance in terms of producing O_2_•^−^ and H_2_O_2_ [90]. These agents can give rise to the •OH via the Fenton reaction and, therefore, NOX enzymes also have been implicated in disease states [91]. XO and MAO are bound to the outer mitochondrial membrane and are free in the cytosol [92,93]. Their generation of ROS increases with age and MAO activity; in particular, it contributes to a major neurodegenerative disease in the aged, i.e., Parkinson disease, in which the dopaminergic neurons of the pars compacta are destroyed [94,95]. 

The cytosolic peroxisome plays a major role in not only the production of H_2_O_2_ but also its detoxification [96]. Disturbances in the redox balance in this organelle promote cellular senescence due to elevated H_2_O_2_ [97]. Additionally, lysosomes release free metal ions such as iron which, via the Fenton reaction, promote •OH formation that advances oxidative stress [98].

While ROS are produced in many portions of every cell, the current review is mainly concerned with mitochondrial ROS (mtROS) and how melatonin functions to combat their toxicity. As will be further elaborated in a later section of this report, melatonin is in especially high concentrations in mitochondria [28], likely due to its rapid uptake [99] and synthesis in this organelle [100,101,102]. The indole is also present in other subcellar compartments but in lower concentrations than in the mitochondria [28]. 

The subcellular distribution of melatonin has been sparingly investigated. Thus, while the differential concentrations of melatonin in various subcellular compartments have been described in a number of cells in different organs [28,103], many cell types have yet to be examined in this regard. Nevertheless, there are numerous reports that support melatonin′s ability to protect molecules in the inner mitochondrial membrane from the toxicity of ROS [104,105,106]. Since the very high reactivity and extremely short half-life of some ROS, e.g., •OH, preclude its transport from where it is produced, if melatonin is to counteract its action in mitochondria, melatonin must be in the immediate vicinity of where the •OH is generated [107,108,109,110,111,112].

## 4. Melatonin and Its Metabolites: Determinants of Oxidative Stress

That melatonin is highly effective in reducing oxidative damage is illustrated in an extensive list of reports published in the last 25 years [113,114,115,116,117]. Mechanistically, the means by which melatonin neutralizes ROS is also well described and includes direct scavenging actions [30,47,118,119,120,121] and indirect processes by which ROS is enzymatically converted to less harmful species [14,107,122,123,124]. In mitochondria, the enzymatic dismutation of O_2_•^−^ involves the stimulation of SIRT3 by melatonin; this leads to the deacetylation and activation of SOD2, thereby limiting oxidative damage to this vital organelle [125,126,127]. The action of melatonin at the mitochondrial level is consistent with its designation as a mitochondria-targeted antioxidant [14,71].

**Figure 3:**
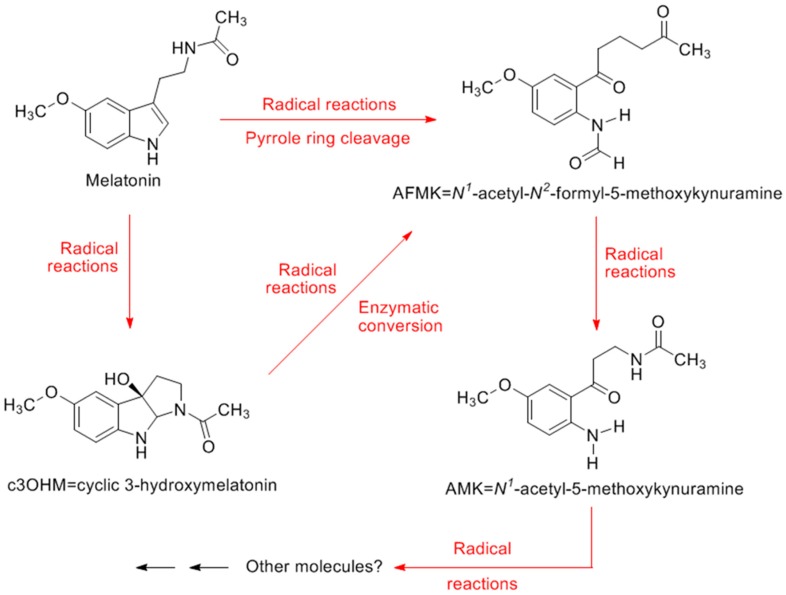
The structure of melatonin and some of its metabolites that have been shown to detoxify reactive oxygen and reactive nitrogen species. Additionally, some of these have other actions that enhance their ability to reduce oxidative stress, e.g., chelation of transition metal ions, promotion of antioxidant enzymes, inhibition of pro-oxidant enzymes, reducing electron leaking from the respiratory chain complexes, etc. Also shown is the sequence by which these metabolites are formed. This sequential formation of metabolites from melatonin, along with their ability to scavenge radicals, is referred to as melatonin′s antioxidant cascade.

While melatonin very effectively reduces oxidative stress in all experimental and clinical settings in which it has been tested [87,128,129,130,131], it has an advantage over other antioxidants, since not only is melatonin a scavenger of toxic species, but several of its products are as well [132,133] (Figure 3). In what is referred to as melatonin's antioxidant cascade, after melatonin interacts with a toxic species it is metabolized enzymatically or non-enzymatically to other antioxidants that are equal to or better than melatonin in detoxifying free radicals [134]. These metabolites include cyclic 3-hydroxymelatonin [135], *N*1-acetyl-*N*2-formyl-5-methoxykynuramine (AFMK) [132], *N*1-acetyl-5-methoxykynuramine (AMK) [133], and perhaps others. Thus, whereas classical antioxidants scavenge a single radical, via its cascade of reactions, melatonin detoxifies multiple radical species. This, coupled with its indirect antioxidant actions described above and its ability to bind transition metals [136], causes melatonin to be a potent repressor of oxidative stress. Moreover, a variety of melatonin analogues, which are produced in vivo, also function as antioxidants [137]. Finally, after being damaged by free radicals, melatonin aids in promoting DNA repair [138].

Considering the wide array of complex actions that melatonin seemingly manifests relative to reducing oxidative stress, it is likely that our understanding of how melatonin actually functions in a highly oxidizing environment is wholly incomplete. The mechanisms that have been put forth to explain melatonin′s comprehensive capacity to provide antioxidant protection leads one to surmise that what is being observed are merely epiphenomena of a more basic molecular action of this phylogenetically-ancient molecule. Future research will likely reveal additional actions(s) of this functionally-diverse agent.

## 5. Melatonin in Mitochondria: A Fortuitous Association

A recently published issue of *Cellular and Molecular Life Sciences* (Volume 74, Issue 21, 2017) is solely devoted to describing the multiple actions of melatonin at the mitochondrial level. These actions likely necessitate that melatonin is present in this organelle. In early immunocytochemical studies, we documented that when astrocytes are challenged in vitro with H_2_O_2_, their mitochondria exhibit an enhanced free radical fluorescence [139]; this enhancement is markedly blunted if, in addition to H_2_O_2_, the cells are simultaneously incubated with melatonin; this protection was superior to that provided by vitamin E. Likewise, using multiple fluorescence imaging microscopy, we observed that melatonin also protects mitochondria from elevated mitochondrial Ca^2+^ (mCa^2+^) stress [140,141]. Thus, melatonin completely attenuated mROS induced by mCa^2+^ due to exposure to ionomycin. Also, melatonin prevented mCa^2+^-mediated mitochondrial permeability transition (MPT), indicating that melatonin may directly target the MPT. When astrocytes were treated with cyclosporine A, a MPT inhibitor, melatonin reduced Ca^2+^-induced cellular apoptosis, showing that melatonin also had actions beyond the MPT. 

Many studies during the last two decades have further defined the critical role that melatonin has in maintaining the optimal physiology of the mitochondria [14,30,87,106,115,142,143,144]. The beneficial actions of melatonin at the level of the mitochondria are apparent in reference to quenching free radicals, reducing oxidative stress, limiting mitochondria-related apoptosis, maintaining the efficiency of the respiratory chain complexes, and ensuring ample ATP production [145,146,147]. Moreover, these regulatory actions are not unique to a single cell type but rather are applicable to every cell, plant, and animal that has mitochondria. 

The remarkable ability of melatonin to preserve mitochondrial function implies that it gets into this organelle in sufficiently high concentrations to protect them from dysfunction under the most extreme oxidizing conditions. There is, however, limited information on the levels of melatonin in subcellular compartments. The one study that made such measurements indicates that, at least in brain cells, melatonin concentrations in mitochondria far exceed those in the blood [28]. While higher than blood levels were also estimated in hepatocyte mitochondria, they were significantly lower than in brain cells. This great difference between these tissues could relate to the much higher concentrations of melatonin in the cerebrospinal fluid (CSF) [148,149], to which brain cells are exposed [150], relative to the much lower levels of melatonin in the blood, to which hepatocytes are exposed. Alternatively, it could relate to the high metabolic demands of neurons versus liver cells. Studies related to the immunocytochemical localization of melatonin at the subcellular level certainly suggest that melatonin quickly enters cells and has ready access to mitochondria [139,140]. The evidence related to the mechanisms by which melatonin may pass through cell membranes was recently reviewed by Mayo and coworkers [151].

Since melatonin is highly lipid soluble, it has often been assumed that it enters cells by simple diffusion. Under detailed examination, however, its passage through the plasma membrane seemed to be dependent on the presence of a protein that was identified as a glucose transporter, GLUT 1 [152]. Docking studies also prompted the conclusion that melatonin′s entrance into the cell was related to the GLUT 1 transporter. This process was slower than expected, however, and did not provide information on how it may contribute to levels of melatonin in mitochondria.

**Figure 4:**
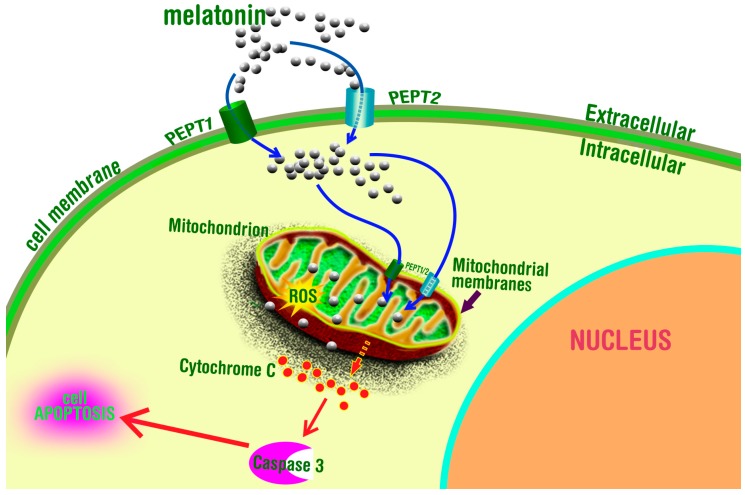
The oligopeptide transporters, PEPT1/2, have recently been reported to be present in mitochondrial membranes. These transporters are believed to move melatonin into mitochondria against a gradient. This may explain the much higher concentration of melatonin in mitochondria than in other subcellular compartments. Moreover, high melatonin levels in mitochondria would be consistent with the marked ability of this antioxidant to protect these organelles from free radical damage as it occurs during aging and as a result of diseases of aging that have a free radical component.

The most complete description of the means by which melatonin enters mitochondria against a gradient is provided by the recent study of Huo et al. [99]. Using two human cancer cell lines (PC3 and U118), this group tested whether either the organic ion transporter, OAT3, or the oligopeptide transporters, PEPT1/2, facilitated melatonin′s transfer through the mitochondrial membranes. The study revealed that the OAT3 transporter was not related to melatonin’s movement into the mitochondria. On the contrary, however, melatonin transport into this organelle was facilitated by PEPT1/2. Docking analysis studies showed that the ability of melatonin to bind to PEPT1/2 related to their low binding energy and optimal binding configuration given that melatonin readily embedded in the active site of the transporters and nestled in the cavity in three dimensional space [99]. Melatonin uptake through PEPT1/2 was linear and its uptake was saturable during prolonged incubation. When Bes, a competitor for the receptors, was added to the incubation medium, melatonin uptake was significantly reduced. The optimal pH for the movement of melatonin through the transporter was 5.5; this is the pH at which PEPT1/2 function maximally. Finally, knockdown of PEPT1/2 expression with siRNAs showed that the amount of melatonin in mitochondria was related to the presence of PEPT1/2 in the membranes of this organelle (Figure 4). Despite the completeness of these studies, the authors left open the possibility that simple diffusion or other yet-to-be-identified transporters may also be involved in the movement of melatonin from the cytosol into the mitochondria. While the findings of Huo et al. [99] are highly significant, whether they will be applicable to other cell types and, in particular, to normal cells (they used cancer cells exclusively) certainly warrants investigation.

In agreement with the observations showing the importance of oligopeptide transporters in hastening the movement of melatonin into mitochondria are studies that compared melatonin with Mito E and Mito Q in terms of their ability to protect cells under extremely high oxidative stress conditions [153]. Mito E and Mito Q are synthetic antioxidants that accumulate in mitochondria at levels up to 500-fold greater than the unaltered antioxidants, i.e., vitamin E and co-enzyme Q10. When rats were treated with two bacterial toxins, lipopolysaccharide (LPS) and peptidoglycan (PepG), which cause severe acute sepsis, there was evidence of substantial malfunction of two organs in which it was assessed, i.e., liver and kidney [153]. In an attempt to protect the organs from damage, the toxin-treated rats were intravenously infused with equimolar concentrations of either Mito E, Mito Q, or melatonin. These molecules had broadly similar protective actions in terms of lowering oxidative stress, preserving mitochondrial respiration, depressing circulating interleukin-6 levels, and protecting against organ dysfunction, as indicated by blood levels of alanine aminotransferase (liver) and creatinine (kidney). In some cases, although it was not proven statistically, melatonin appeared superior to the synthetic molecules, Mito E and Mito Q. The ability of melatonin to protect against experimental [87,154,155] and clinical [156] sepsis is well documented. On the basis of their findings, Lowes and colleagues [153] proposed the use of melatonin over Mito E or Mito Q in clinical trials designed to attenuate mitochondrial oxidative stress and cellular dysfunction. Considering that Mito E and Mito Q concentrate in the mitochondria up to 500 times greater than the levels in the blood, along with the fact that melatonin provides equal or better protection, it can be assumed that the indole was also in high concentrations in mitochondria.

Given what is known about the seemingly high levels of melatonin in mitochondria and the ability of this compartment to avidly take up the indole, the speculation that melatonin is targeted to the mitochondria is certainly justified [30,57,71,157]. Elevated mitochondrial melatonin levels would certainly be advantageous, since these organelles produce the bulk of the damaging free radicals that most cells generate. Moreover, since they are the site of origin of the majority of the ATP, they are absolutely vital to the survival of a cell. This being the case, it should not be unexpected that mitochondria would have access to a highly potent antioxidant, such as melatonin. 

**Figure 5:**
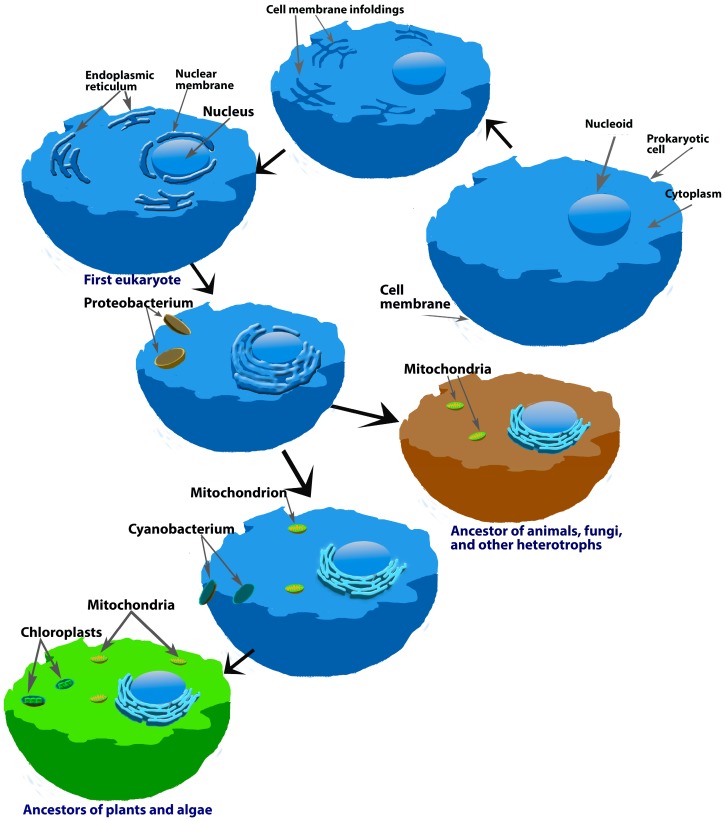
This is an illustration of what is referred to as the endosymbiotic theory for the origin of mitochondria (and chloroplasts) and why these organelles in present-day eukaryotes likely have the ability to produce melatonin. A couple of billion years ago, prokaryotes phagocytized proteobacteria, which are known to synthesize melatonin; these bacteria were initially digested and used as nutrition. During evolution, the ingested bacteria eventually developed a mutually-beneficial symbiotic relationship with the cells that ingested them and they evolved into mitochondria. When they did so, the evolved mitochondria retained the ability to produce melatonin (brown image). As a result, present-day eukaryotic cells synthesize melatonin as shown in several reports cited in the current review. Likewise, some of the same prokaryotes also engulfed photosynthetic, melatonin-producing bacteria which evolved into chloroplasts of plant cells (green image); chloroplasts also have been shown to be involved in melatonin synthesis. Since plant cells have both chloroplasts and mitochondria may explain why plants generally have higher cellular concentrations of melatonin than do animal cells, which only have mitochondria.

The detection of melatonin in the earliest-evolved organisms, i.e., bacteria [11,12,158], along with theory that mitochondria/chloroplasts evolved from bacteria that were initially ingested by prokaryotes as food [159] (Figure 5), prompted the speculation that mitochondria/chloroplasts retained the melatonin-forming ability of their bacterial precursors [100]. This would be a very fortuitous arrangement given the fact that, as already mentioned herein, mitochondria have a propensity to produce an abundance of free radicals as a byproduct of oxidative phosphorylation [57,71,142,157]. Recently published data supports the high likelihood that both mitochondria [71,100,101,102,160] and chloroplasts [161,162] are in fact sites of melatonin synthesis.

In reference to melatonin synthesis by mitochondria, He and colleagues [160] isolated these organelles from mouse oocytes and showed, immunocytochemically, that they express the rate limiting enzyme in melatonin synthesis, serotonin *N*-acetyltransferase. When purified mitochondria were incubated with serotonin for 1 hour, the melatonin concentration in the culture medium rose dramatically. Since mitochondria were the only organelles in the incubation medium, it was assumed the rise in melatonin in this fluid was a consequence of its sustained production by mitochondria. As physiological evidence that melatonin was present in mitochondria, the amount of melatonin correlated with several parameters that were improved in these organelles (increased mtDNA copies, elevated ATP, enhanced mitochondrial membrane potential, etc.).

The most complete documentation of mitochondria being the site of melatonin synthesis comes from a recent study of Suofa et al. [102]. They reasoned that since melatonin levels are highly elevated in brain mitochondria [28], that it may also be produced in these organelles. With this rationale and using rat forebrain non-synaptosomal mitochondria, they tested whether the melatonin-forming enzymes, i.e., arylalkylamine *N*-acetyltransferase (AANAT) and *N*-acetylserotonin-*O*-methyltransferase (ASMT), are also present. When these constituents were measured, the authors found this to be the case. Additionally, 14-3-3 was also located in the mitochondria (and in the cytosol); 14-3-3 is a chaperone that is present in the pineal gland [62] and protects AANAT from degradation and improves its affinity for serotonin, the substrate for the enzyme [163]. Digestion of the outer mitochondrial membrane with a combination of proteinase K and digitonin (which left the inner mitochondrial membrane intact) revealed that the remaining mitochondrial fraction retained the AANAT and ASMT activities, suggesting their location in the mitochondria matrix. Unlike in the pineal gland, mitochondrial AANAT activity did not vary over a light:dark cycle. When AANAT was knocked out in mouse neuroblastoma cells (N2a), mitochondria exhibited a much higher degree of oxidative stress in response to oxygen/glucose deprivation; this is consistent with the absence of melatonin, since it is a potent inhibitor of oxidative damage [71]. Finally, when purified mouse forebrain mitochondria were incubated with deuterated (D4) serotonin along with ATP and respiratory substrate, they quickly formed D4-*N*-acetylserotonin and D4-melatonin. 

The importance of the findings suggesting that mitochondria produce their own melatonin cannot be over emphasized. Mitochondria are a major site of free radical generation and, therefore, oxidative damage. Malfunctions of mitochondria have numerous debilitating consequences in terms of cellular loss, organ dysfunction, and organismal decline (see below).

In view of the data reported, we predict that melatonin will be inducible in mitochondria as has been shown in plants [21,26,164,165,166]. We suspect the stimulus for the compensatory rise in mitochondrial melatonin production will be the quantity of free radicals being formed in these organelles or the amount of oxidative damage being sustained. When the dinoflagellate *Lingulodinium polyedrum* (nee, *Gonyaulax polyhedron*) is exposed to a reduced ambient temperature, a situation that augments free radical generation in these unicells, melatonin levels in these organisms rise dramatically [167]. The increase is likely related to the de novo synthesis of the indole. As noted above, a compensatory elevation in melatonin levels is also a common feature of land plants when they experience any of a number of stresses [21,26,164,165,166].

## 6. Melatonin, Oxidative Stress, and Aging

That morphological, functional, and molecular deterioration occurs with increasing age is indisputable for all living organisms; however, the rate at which different taxa degenerate varies widely. A reliable explanation for these differences is yet to be fully defined and, in fact, aging mechanisms of a given species likewise constitute a major point of scientific debate.

The subcellular organelle most frequently implicated in terms of determining the rate of aging is the mitochondrion [168,169]. Moreover, it is the elevated free radicals that they produce that theoretically help to explain their age-related dysfunction. The free radical theory of aging has persisted for more than 50 years [170,171], but the evidence supporting it is still not beyond dispute [165,168].

Since free radicals obviously cause molecular disfigurement and functional decay, and given that they are abundantly created in mitochondria, molecules that either efficiently quench a variety of ROS species or reduce their production, especially in mitochondria, may be useful as a means to slow the rate of aging and reduce age-related diseases [172,173]. To enhance their entry into mitochondria, industry has synthesized synthetic antioxidants [174,175] that do accumulate up to 500-fold (greater than the natural antioxidant) in the mitochondrial matrix. Despite this, these modified radical scavengers are no better than melatonin in fending off ROS-mediated damage [138]. Because of this and for other reasons, interest is the role of melatonin in aging processes has been a topic of interest for more than two decades [176,177,178].

In 1999, we [179] reported that surgical removal of the pineal gland from young rats caused a more rapid accumulation of oxidatively-damaged molecules in multiple tissues when the animals reached 25 months of age. Pinealectomy deprives animals of melatonin that are normally secreted by the pineal, but not from other organs. Thus, the animals were not devoid of melatonin but they presumably were relatively melatonin deficient compared to pineal-intact animals. The findings could mean that the partial loss of the antioxidant, melatonin, was responsible for the accelerated oxidative damage measured later in life, i.e., the animals aged more rapidly. The data, however, must be considered in light of another action of melatonin not related to its free radical scavenging actions. Since the circadian rhythm of melatonin feeds back onto the master biological clock, SCN, to aid in the synchronization of rhythms throughout the organism [180], the circadian disruption due to pineal removal may have led to the excessive accumulation of oxidative damage [181,182]. The loss of the endogenous melatonin rhythm is always accompanied by some total body circadian dysregulation [183]. Twenty-four hour rhythms are also fundamental properties of mitochondria with both their morphology and physiology being periodic [184]. Systematic, as well as intrinsic, cues probably drive these mitochondrial cycles [87]. Thus, when examining the actions of melatonin on molecular aspects of aging, and diseases associated with advancing age, it is important to note that melatonin may have positive actions beyond its ability to squelch oxidative stress, i.e., function as an antioxidant. The multiple actions of melatonin may explain why melatonin is better than conventional antioxidants in preserving optimal cell physiology and seemingly improving health.

Considering the multiple critical functions of mitochondria, it is not unexpected that they would be the focus of research related to aging. The diverse functions of this organelle include optimizing oxidative phosphorylation (OXPHOS), which culminates in ATP production; during this process oxygen-based radical and non-radical products are formed that, in the long term, damage the mitochondria. This organelle also participates in metabolic and signaling pathways including the regulation of apoptosis. The mitochondria also possess their own genetic material [mitochondrial (mt)DNA]. mtDNA encodes some of the proteins that are components of the respiratory complexes. Given the inadvertent production of ROS during OXPHOS, mtDNA is readily damaged, leaving it to form proteins of the respiratory chain that are flawed; when this occurs, the complexes become inefficient and produce higher numbers of damaging free radicals. These processes then become a progressively increasing cycle of destructive reactions leading to accelerated deterioration of mitochondrial physiology, which contributes to the aging phenotype [185,186].

Presumably, both because of their ability to uptake [99], as well as to synthesize [102], melatonin, the mitochondria contain sufficiently high concentrations of the indole to resist the mitochondrial melee initiated by ROS. There are numerous experimental data documenting melatonin’s ability to defer mitochondrial mutilation and dysfunction resulting from the excessive production of ROS due to the inefficient transfer of electrons between the respiratory complexes [84,125,128,130,144,153,187,188,189,190,191,192]. Melatonin likely achieves its protective effects because of its scavenging activities, as well as those of its metabolites [14,15,30,46,47,68,71], in addition to its indirect actions in the activation of mitochondria-located SOD2; in this case, melatonin stimulates SIRT3 activity, which prompts the deacetylation and activation of SOD2, thereby reducing the oxidative burden of the mitochondria [126].

**Figure 6:**
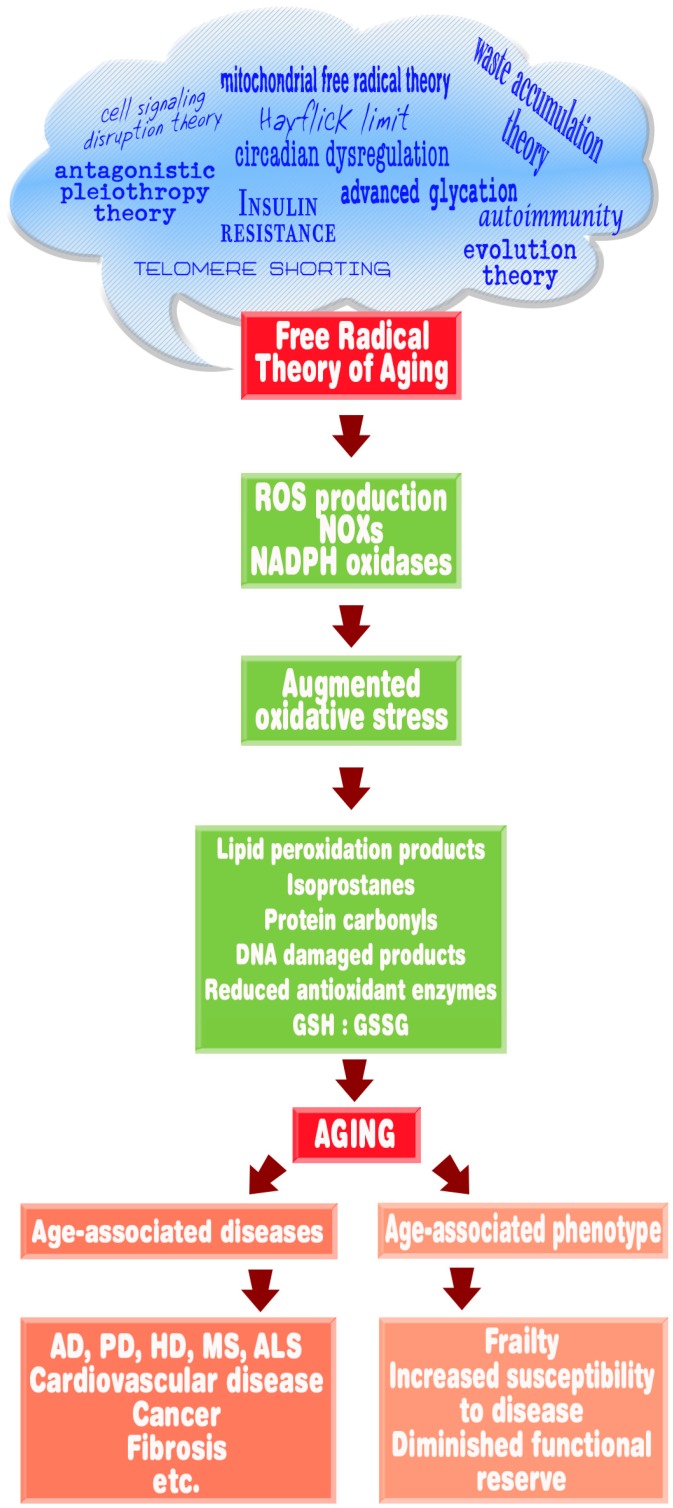
This figure is a flow diagram that links free radicals and the associated oxidative damage with the progression of the aging phenotype and the onset and development of age-related diseases. The cloud at the top lists many of the iterations of the free radical theory of aging that have been introduced over the last 60 years. In the current review, we discuss the evidence that melatonin could be relevant to the processes summarized. ROS = Reactive oxygen species; AD = Alzheimer disease; PD = Parkinson disease; HD = Huntington disease; MS = Multiple sclerosis; ALS = amyotrophic lateral sclerosis.

Pineal-derived blood (and likely CSF) melatonin concentrations often wane as individuals age, thereby reducing the ability of melatonin to stabilize circadian rhythms and lowering its radical quenching ability [179,193]. Blood concentrations of melatonin diminish since its source, the pineal, loses its ability to synthesize it [194,195]. Hence, the drop-in melatonin is one of perhaps a number of factors that contributed to elevated oxidative injury in the elderly, including an increased incidence of diseases that have a significant free radicals component (Figure 6). 

While it has been assumed that peripheral organ melatonin levels also drop with age due to a reduced local production, the evidence for this is scanty [196]. Nevertheless, we surmise that the total melatonin load is significantly greater in young animals (including humans) compared to old members of the species, and that the levels drop at a rather consistent rate in all organs as individuals age. The persistent shrinking levels of melatonin throughout life presumably contribute to the slow decline of organ function characteristic of aging. Certainly, the published literature is saturated with studies showing that restoring the diminished melatonin levels by supplementing them delays or restores physiological degeneration in old animals [84,85,87,95,104,197,198,199,200,201] and humans [88,202,203,204,205,206].

Paradies and colleagues [104] recently reviewed the expansive literature related to the degeneration of mitochondrial physiology during aging along with the capacity of melatonin to revitalize the function of these critically-important organelles. A reduction in the functional efficiency of the mitochondrial electron transport chain, including a diminished ability to generate ATP, has been repeatedly documented [142,145,207,208,209]. As discussed in their review, melatonin′s confirmed beneficial actions during aging very likely stem, in a major way, from its ROS scavenging activity in the mitochondrial matrix and intermembrane space [71]. In doing so, melatonin reduces oxidative damage, cardiolipin oxidation, MPTP opening, cytochrome c release, and cellular apoptosis. Melatonin may also have a direct action on the MPTP to reduce pore opening [210]. There is essentially universal agreement that shielding mitochondria from age-associated dysfunction would slow the processes of aging generally and especially the development of certain age-related diseases, e.g., neurodegenerative conditions [178,183,187,192,201,202]. Considering these data, melatonin′s multiple beneficial actions at the mitochondrial level seem to justify the conclusion that this endogenously-produced and exogenously-acquired indoleamine has a role in determining the rate at which both plants [211,212] and animals [178] age.

## 7. Concluding Remarks

Based on the literature surveyed in this review, it might be assumed that the routine long term use of supplemental antioxidants would aid in deferring aging and in delaying the onset or progression of age-associated diseases. However, taking conventional antioxidants, e.g., vitamins C or E, even in large quantities to improve performance or delay fragility has certainly not been unequivocally successful [213,214]. In contrast, the most frequent recommendation to support the anti-aging goals is dietary intake rich in multiple antioxidants and other nutrients [215]. 

Considering these observations and recommendations, it is reasonable to question whether treatment with melatonin, a molecule with obvious antioxidant activities, would yield results different than those provided (or not provided) by the vitamin antioxidants. When it functions to reduce oxidative damage, it is a more general antioxidant and displays multiple means to limit free radical damage [15,30,47,71,120,121]; this is a feature generally not shared by the vitamin antioxidants that have specific actions. Moreover, melatonin targeting to and synthesis in mitochondria likely affords it protective means not shared by the vitamin antioxidants. Thus, melatonin is an unconventional antioxidant with uncommon actions, some of which are probably yet to be identified.

It may also be futile to expect that the use of a single molecule would defer aging considering the complexity of the aging process [216]. Yet, interest in sole treatments such as metformin [217,218] is in vogue, and this molecule has generated a clinical trial [219]. Only two reports known to the current authors that compared the benefits of metformin relative to those of melatonin have been published [220]. In the first of these reports, the ability of melatonin and metformin, alone or in combination, to reduce testicular damage due to torsion-mediated ischemic injury was compared [221]. Histologically, these agents were equally effective in preserving spermatogenic activity and providing antioxidant protection to the gonads. Combining the two treatments, however, did not further improve the parameters measured in the ischemic testes. In the second report, melatonin and metformin were compared relative to their ability to limit oxidative stress to the heart of rats with mammary tumors [220]. Each molecule individually reduced free radical-mediated lipid and protein damage while promoting antioxidant enzyme activities, although melatonin was generally more effective in protecting against oxidative stress. The outcome of the latter comparison prompted the authors to conclude that melatonin has significantly greater antioxidant activity than metformin at the level of the heart. Experimental treatments that include both melatonin and metformin may be timely and could yield useful data for the design of clinical studies with an interest in modifying aging processes.

An important take-home message from this review is that melatonin should not be thought of as a regular antioxidant; the published data, which is extensive, indicates otherwise. The mere fact that it is both consumed in the diet and exogenously produced, perhaps in every mitochondria/chloroplast-containing cell of every living organism, makes melatonin unique. Additionally, the fact that melatonin is so closely associated with mitochondria should make it of significant interest in any study in which the endpoints include deferring the onset of diseases, improving the quality of life, or prolonging longevity.
